# High resolution diffusion-weighted imaging with readout segmentation of long variable echo-trains for determining myometrial invasion in endometrial carcinoma

**DOI:** 10.1186/s40644-020-00346-7

**Published:** 2020-09-21

**Authors:** Mengnv Xie, Zhen Ren, Dujun Bian, Dan Li, Li Yu, Fang Zhu, Rui Huang, Zhibang Zhang, Suye Suye, Chun Fu

**Affiliations:** 1 Department of Obstetrics and Gynecology, The Second Xiangya Hospital, Central South University, Changsha, No.139 Renmin Road, Changsha, Hunan 410011 PR China; 2 Department of Radiology, The Second Xiangya Hospital, Central South University, Changsha, Hunan PR China

**Keywords:** Magnetic resonance imaging, RESOLVE DWI, Myometrial invasion, Endometrial carcinoma

## Abstract

**Background:**

We assessed the image quality of endometrial cancer lesions by readout segmentation of long variable echo-trains (RESOLVE) diffusion-weighted imaging (DWI) compared with that by single-shot echo-planar imaging (SS-EPI) DWI, aimed to explore the value of RESOLVE DWI for determining myometrial invasion and clinical stage in endometrial cancer.

**Materials and methods:**

From April 2017 to March 2018, a total of 30 endometrial cancer patients (mean age 52.8 ± 9.0 years), who had undergone RESOLVE DWI and SS-EPI DWI, were included in the study. The image quality of endometrial carcinoma by two kinds of DWI scanning methods was compared qualitatively and quantitatively. The Spearman rank correlation test was used to assess the correlation of qualitative image quality scores between two readers. The accuracy of two DWI methods in detecting myometrial invasion and staging of endometrial carcinoma was calculated according to postoperative pathological results. The indexes were analyzed including sensitivity, specificity, accuracy, positive predictive value (PPV), and negative predictive value (NPV).

**Results:**

The qualitative score of RESOLVE DWI group was superior to SS-EPI DWI group in every aspect of five aspects (all *P* < 0.001). Interobserver agreement of depiction was good or excellent in two DWI sequences. Signal to noise ratio and contrast to noise ratio values in RESOLVE DWI group were both higher than those in SS-EPI DWI group (*P*<0.001). No statistical difference of apparent diffusion coefficient value was observed between two DWI groups (*P* = 0.261). The specificity, accuracy, PPV, and NPV of estimating myometrial invasion by RESOLVE DWI in three cases (intramucosal lesion, <50% superficial invasion and ≥ 50% deep invasion) were all higher than those by SS-EPI DWI for endometrial carcinoma. Especially RESOLVE DWI was valuable in judging <50% superficial invasion (95%CI:0.586, 0.970). No significant difference in accuracy staging was between the two DWI groups (*P* = 0.125).

**Conclusion:**

RESOLVE DWI can provide higher quality images of endometrial carcinoma than SS-EPI DWI. The high-quality images are helpful for precise assessment of myometrial invasion in endometrial cancer.

## Background

Endometrial cancer is one of the most common malignant tumor threatening to female health [1]. The incidence of endometrial cancer is second-ranked in female genital tract tumors only lower to cervical cancer in China. Preoperative evaluation of endometrial cancer is very important for tailoring treatment. The purpose of preoperative evaluation is mainly to evaluate intrauterine lesions, with emphasis on the depth of myometrial invasion and the extent of local spread [2]. Several imaging methods have been used to evaluate intrauterine lesions for endometrial cancer. Which method is more suitable for the evaluation of endometrial cancer is still in the exploration.

Magnetic resonance imaging (MRI) has been widely accepted for the evaluation of myometrial invasion and extrauterine tumor spread owing to its superior contrast resolution and excellent soft tissue differentiation [3]. However, conventional MRI sequences like T1-weighted imaging (T1WI) and T2-weighted imaging (T2WI) which lacks specific parameters regarding tumor microstructure or biological information have limitations in assessing myometrial invasion. Functional MRI techniques like diffusion-weighted MRI (DW-MRI) and dynamic contrast-enhanced MRI (DCE-MRI) can improve the accuracy of myometrial invasion. Diffusion-weighted imaging (DWI) is commonly used in pelvic scanning. Multiple studies of endometrial cancer have shown that DWI images and apparent diffusion coefficient (ADC) values are helpful to evaluate the depth of myometrial invasion [4–8]. The accuracy and sensitivity of assessing myometrial invasion by DWI are higher than those by DCE-MRI in endometrial cancer [9].

DWI has been used as an adjunct to conventional MRI in the evaluation of endometrial cancer lesions by providing quantitative information about water molecular diffusion, but its disadvantage is leading degradation of image quality [10]. Therefore, the improvement of the scanning sequence has been the key to DWI image quality. Single-shot echo-planar imaging (SS-EPI) is a commonly used scanning sequence of DWI, but artifacts and blurring effects may occur due to the long time of data acquisition and accumulated phase errors [11]. Readout-segmented echo-planar imaging (RS-EPI) is a new DWI scan sequence, which can shorten the sampling time and reduce the accumulation of phase errors in the direction of phase encoding so that a high-resolution DWI images can be generated [12]. DWI using RS-EPI is also called readout segmentation of long variable echo-trains diffusion-weight imaging (RESOLVE DWI) (Response comment 1). RESOLVE DWI has been used in the brain, spine, breast, and kidney imaging [13–16]. Its application in pelvic is mainly concentrated in rectum, prostate and bladder imaging [17–19].

Therefore, this study aimed to apply RESOLVE DWI to endometrial cancer. The study had two purposes. The primary was to compare the image quality of endometrial cancer lesions with RESOLVE DWI and SS-EPI DWI. The secondary was to evaluate the value of RESOLVE DWI in determining myometrial invasion and clinical stage of endometrial cancer.

## Materials and methods

### Patients

**Fig. 1:**
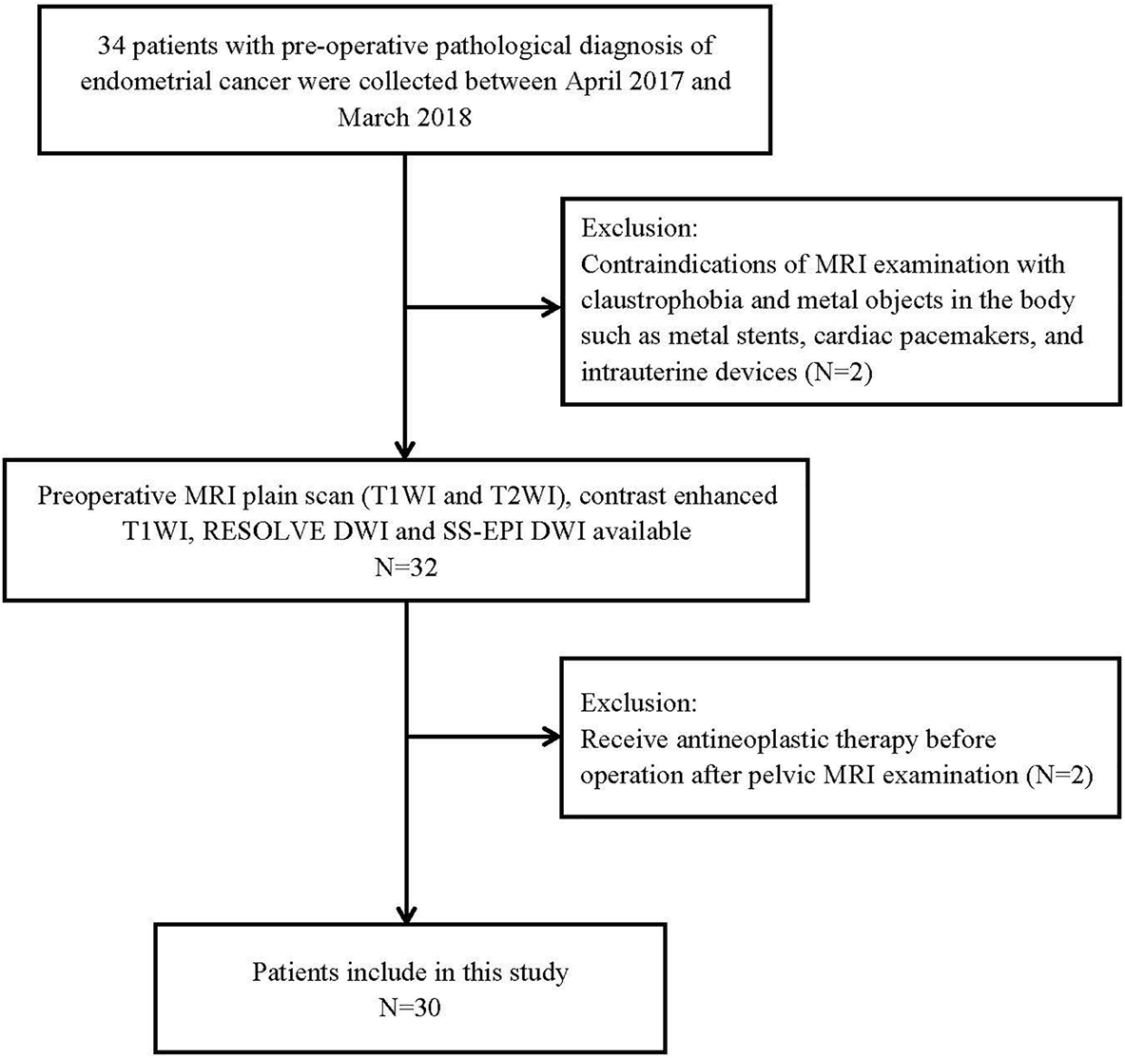
Illustration of the flow diagram of included patients

The comparative study of two scanning methods was approved by the Institutional Review Board. Written informed consent was obtained from all patients before treatment. 34 patients with pre-operative pathological diagnoses of endometrial cancer were collected between April 2017 and March 2018. Four patients were excluded: (1) contraindications of MRI examination with claustrophobia and metal objects in the body such as metal stents, cardiac pacemakers, and intrauterine devices (*n* = 2); (2) receiving antineoplastic therapy before operation after pelvic MRI examination (*n* = 2). 30 patients (mean age, 52.8 ± 9.0 years; range, 32–75 years) were finally included in our study (Fig. 1). Each patient received T1WI, T2WI, contrast-enhanced T1WI, RESOLVE DWI, and SS-EPI DWI.

### MRI protocol

All MRI examinations were performed at 3.0 T MRI scanner (Magnetom Skyra, Siemens, Erlangen, Germany). The coil specifications were standard 16-channel phased-array body coil and 32-channel phased-array spinal coil. The scan ranges from the upper margin of the ilium to the symphysis pubis. All patients received MRI examinations in the non-menstrual period. Each patient had a fasting for 6 h before examination and kept the bladder adequately filled.

Scan sequences contained turbo spin-echo (TSE) T1WI, TSE T2WI, contrast-enhanced TSE T1WI, RESOLVE DWI, and SS-EPI DWI. TSE T1WI was acquired in axial planes. Fat suppressed TSE T2WI were acquired in three orthogonal planes (axial, sagittal, and coronal). Immediately after bolus injection of Gadopentetate Dimeglumine (Gd-DTPA) with a dose of 0.1 mmol/kg (Magnevist, Schering, Berlin, Germany), fat-suppressed TSE T1WI were obtained in the axial, sagittal, and coronal planes. All patients underwent SS-EPI and RS-EPI DWI scanning of pelvic cross-section, respectively. Meanwhile, images reconstruction of diffusion sensitive gradient b value 0 s/mm^2^ and 1000 s/mm^2^ was completed. The protocol parameters were shown in Table 1.

**Table 1:** Imaging parameters for T1-,T2- and contrast-enhanced T1-weighted MRI and SS-EPI, and RESOLVE DWI sequences

Parameters	T1WI	Fat-suppressed T2WI			Contrast-enhanced T1WI			SS-EPI DWI	Resolve DWI (RS-EPI) DWI
Scan plane	axial	axial	sagittal	coronal	axial	sagittal	coronal	axial	axial
Slice thickness, mm	4	4	4	4	4.5	4	4.5	4	4
Slice gap, mm	0.4	0.4	0.4	0.4	0.45	0.4	0.45	0.4	0.4
TR, ms	500	5460	4450	5460	507	582	500	6800	5700
TE, ms	12	87	87	87	12	9	9	113	78
Matrix	320 × 320	320 × 320	320 × 320	320 × 320	320 × 320	320 × 320	320 × 320	192 × 192	200 × 200
FOV, mm	300 × 300	300 × 300	280 × 280	280 × 280	240 × 240	240 × 240	260 × 260	300 × 300	300 × 300
Flip angle	160°	148°	156°	148°	160°	180°	180°	–	–
b value, s/mm^2^	–	–	–	–	–	–	–	0; 1000	0; 1000
Average	2	2	2	2	2	2	2	2	2
Scan times	2min14s	2min37s	2min44s	2min14s	2min52s	3min32s	3min14s	2min9s	4min59s

*MRI* magnetic resonance imaging, *SS-EPI* single-shot echo-planar imaging, *RESOLVE* readout segmentation of long variable echo-trains, *DWI* diffusion-weighted imaging, *T1WI* T1-weighted imaging, *T2WI* T2-weighted imaging, *RS-EPI* readout-segmented echo-planar imaging, *TR* time of repeat, *TE* time of echo, *FOV* field of vision, *min* minutes, *s* second

### Image analysis

All the measurements and evaluations were conducted by Siemens 3.0 T Skyra MR work station (version syngo MR D13). The MRI images were reviewed independently by two senior radiologists respectively with 10 years and 12 years of working experience and were blinded to the histopathology reports. Any discrepancies were resolved by consensus. The analytic content was as follows: qualitative and quantitative assessments of image quality, the depth of myometrial invasion, and the preoperative stage.

### Qualitative evaluation of image quality

Based on the related literature, the study firstly analyzed the quality of MRI images from four aspects (b 1000 s/mm^2^). These four aspects were geometric distortion, image blurring, ghosting artifacts, and lesion conspicuity [20]. The score degree of each aspect was divided into a 6-point scale (ranging from 1 to 6, listed in Table 2) [21]. The scores of the aforementioned four items were further divided into a 3-point scale as follows: for items with a score of 5–6, accumulate 2 points; 3–4 points, accumulate 1 point; 1–2 points, accumulate 0 point. Finally, the radiologist accumulated the scores of four items to get the overall image quality scores. A score of 8 is the best and 0 is the worst.

**Table 2:** Detailed rules for qualitative analysis of image quality

Parameter	Score					
	6	5	4	3	2	1
Geometric distortion	no distortion	probably no distortion	faint severe distortion	partially severe distortion	severe distortion	extremely severe distortion
Image blurring	no blurring	probably no blurring	faint severe blurring	partially severe blurring	severe blurring	extremely severe blurring
Ghosting artifacts	no artifact	probably no artifact	faint severe artifact	partially severe artifact	severe artifact	extremely severe artifact
Lesion conspicuity	outstanding	good	above average	below average	poor	unacceptable
Overall image quality	outstanding	good	above average	below average	poor	unacceptable

### Quantitative evaluation of image quality

**Fig. 2:**
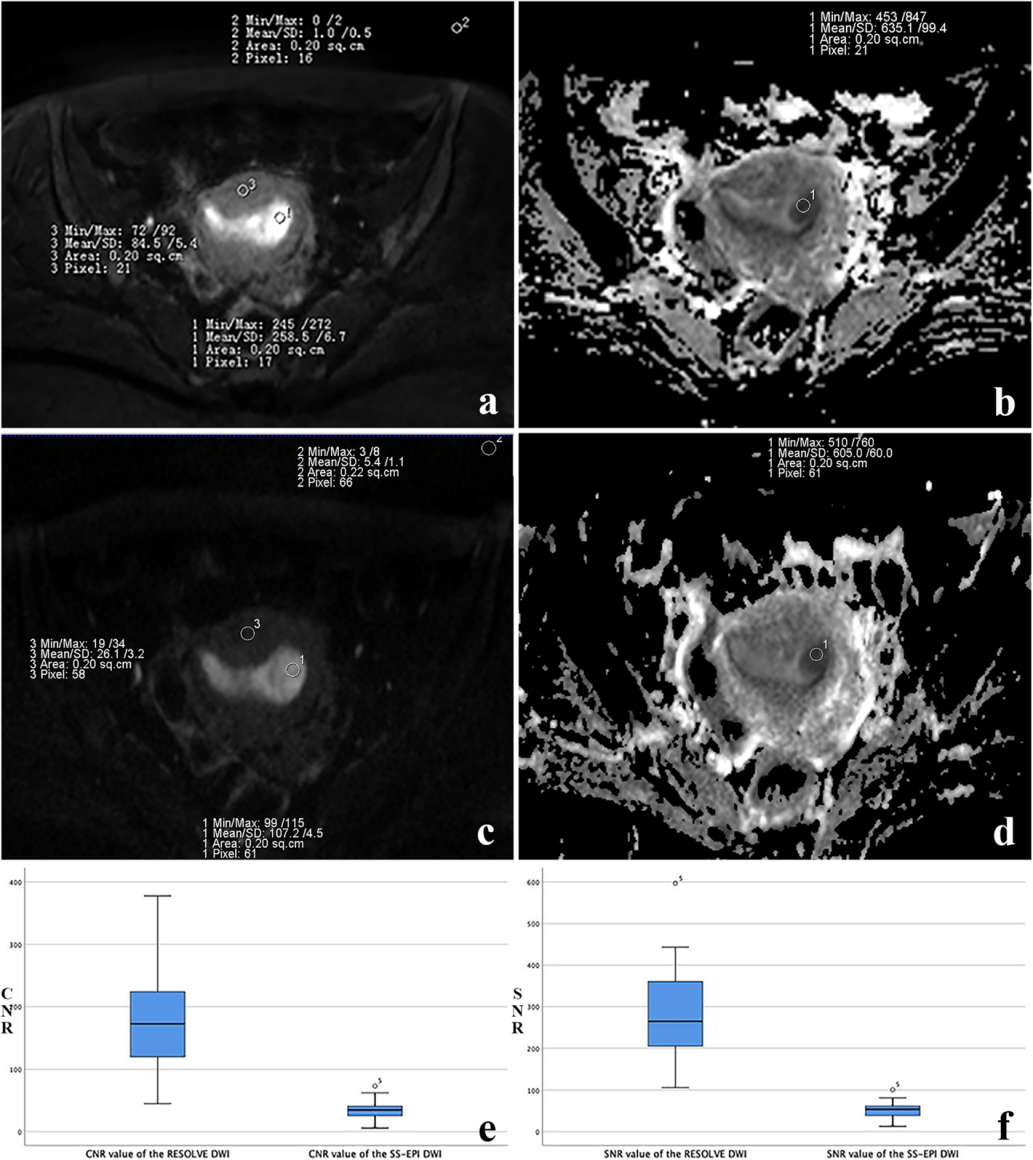
Acquisition value of signal to noise ratio (SNR), contrast to noise ratio (CNR) and apparent diffusion coefficient (ADC) on cross-sectional MRI (b = 1000s/mm^2^). **a** SNR and CNR measurement on readout segmentation of long variable echo-trains diffusion-weight imaging (RESOLVE DWI) image. ROI 1, the signal intensity (SI) of the lesion (SI lesion); ROI 2, SI of background noise (SI background); ROI 3, SI of the myosphere of the uterus (SI myosphere). **b** ADC value measurement of lesion on ADC maps of RESOLVE DWI. ROI 1, the ADC value of the lesion. The mean SNR, CNR and ADC values are 517, 348 and 635.1 × 10^−6^ mm^2^/s, respectively. **c** SNR and CNR measurement on single-shot echo-planar imaging diffusion-weighted imaging (SS-EPI DWI) image. ROI 1, the signal intensity (SI) of the lesion (SI lesion); ROI 2, SI of background noise (SI background); ROI 3, SI of the myosphere of the uterus (SI myosphere). **d** ADC value measurement of lesion on ADC maps of SS-EPI DWI. ROI 1, the ADC value of the lesion. The mean SNR, CNR and ADC values are 97.5, 73.7 and 605 × 10^− 6^ mm^2^/s, respectively. **e** Box plot shows significant differences were found in CNR values between the RESOLVE DWI and DWI group (*P* < 0.05). **f** The SNR values of the RESOLVE DWI group was significantly higher than that of the DWI group (*P* < 0.05)

The quantitative analysis included the measurement of signal to noise ratio (SNR), contrast to noise ratio (CNR) and ADC. The measurement method on RESOLVE DWI is shown in Fig. 2 (b = 1000s/mm^2^). Each index was measured three times for each case, and the average was taken as its final value. SNR was defined by the following formula: SNR = SI (lesion)/SD (background) [22]. SI (lesion) represented the signal intensity (SI) of the lesion. SD (background) referred to the standard deviation of the SI of background noise. The region of interest (ROI) with the minimum size of 10mm^2^ was obtained on the axial sections. All ROIs were put on the same slice on which the maximum area of the endometrial carcinoma shown. The selected ROIs of SI (lesion) were consistent with that of ADC value measurement in the same patient. CNR was defined by the following relationships: CNR = (SI (lesion) –SI (myometrium))/ SD (background) [23]. SI (lesion) and SI (myometrium) represented the SI of the lesion and myometrium, respectively. The ROI of myometrium was selected as the same size and layer of the lesion. ADC was defined by the following formula: ADC = In (SI_0_/SI_1_)/(b_1_-b_0_). b_1_ = 1000s/mm^2^, b_0_ = 0 s/mm^2^. SI_1_ and SI_0_ represented the SI of the lesion at a b value of 1000s/mm^2^ and 0 s/mm^2^, respectively [15]. Freehand ROIs of RESOLVE DWI were carefully matched to the SS-EPI in the same patient.

### MRI appearance of myometrial invasion and preoperative staging of endometrial cancer

T2WI and DWI were evaluated together in order to determine the depth of the myometrial invasion and preoperative stage of endometrial cancer. Simultaneous assessment of the findings of T2WI and DW images was defined as “T2-DWI”. Two radiologists both of whom were blinded to the histopathological reports assessed the T2-RESOLVE DWI and T2-SS-EPI DWI images with 2 weeks apart between two sequences and evaluated the depth of myometrial invasion. Differences in the assessment were resolved by means of consensus. The method of judging the degree of myometrial invasion in the study was described by Nougaret S [24]. A line was drawn along the expected inner edge of the myometrium (endometrium–myometrium junction) on axial oblique images acquired perpendicular to the endometrium. Then, 2 lines are drawn: one measuring the thickness of the entire myometrium an another measuring the maximum tumor extent within the myometrium. The ratio of the 2 lines detailed represents the percentage of myometrial invasion. The depth of myometrial invasion were divided into 2 categories according to the FIGO staging system (2009) (Supplementary Table 1). The presence of myometrial invasion limited to the endometrial cavity or less than 50% was considered to be as IA and the presence of equal to or more than 50% myometrial invasion was considered to be as IB. For more detailed analysis, myometrial infiltration was divided into three cases as followers: intramucosal lesion, less than 50% superficial invasion and equal to or more than 50% myometrial invasion (Supplementary Table 2).

### Reference standard

All patients underwent total hysterectomy, bilateral salpingo-oophorectomy, and pelvic nodal dissection with or without para-aortic nodal dissection. A specialized gynecology/oncology pathologist with 20 years of experience performed the histopathologic examination of all samples. The final histologic result was available for each patient and constituted the reference standard for comparison. Depth of myometrial invasion (superficial invasion, confined to endometrium or inner half of the myometrium; deep invasion, invading the outer half of the myometrium), presence of cervical stromal invasion, and presence of metastasis within the sampled lymph nodes were confirmed microscopically. The staging standard of endometrial cancer patients was the International Federation of Obstetrics and Gynecology (FIGO) 2009 criteria.

### Statistical analysis

All statistics were performed using SPSS 25.0. Differences among the data were considered statistically significant at *P* < 0.05. The SNR, CNR, ADC value and qualitative scores of image quality were compared between the RESOLVE DWI and SS-EPI DWI group respectively. The Paired t-test was used for continuous variables (SNR and CNR). The Wilcoxon’s signed-rank method for non-normal Distribution Variables (ADC and qualitative scores of image quality).

The Spearman rank correlation test was used to assess the correlation of qualitative image quality scores between two readers. The correlation coefficient rho (r_a_) was obtained to compare the degree of correlation as follows: little or no relationship if 0 ≤ r_a_ < 0.2, fair if 0.2 ≤ r_a_ < 0.4, moderate if 0.4 ≤ r_a_ < 0.6, good if 0.6 ≤ r_a_ < 0.8, and excellent if 0.8 ≤ r_a_. The accuracy of myometrial invasion and preoperative staging of endometrial cancer by RESOLVE DWI and SS-EPI DWI was calculated according to pathological results. The difference between the RESOLVE DWI and SS-EPI DWI group was tested by Fisher’s exact probability method. The sensitivity, specificity, accuracy, positive predictive value (PPV), and negative predictive value (NPV) of judgment myometrial invasion by RESOLVE DWI and SS-EPI DWI were also calculated based on pathological results.

## Results

### Clinical features

Of the 30 patients in the study, 16 were postmenopausal with median menopause time 5.5 years (range, 1–20 years). All patients underwent operation within 2 weeks after the MRI examination. The types of operations were summarized in (Supplementary Table 3). The cases of FIGO stage IA, IB, II, IIIA, IIIB and IV were 21, 4, 1, 1, 2, 1, respectively. Five pathological types were described as follows: endometrial adenocarcinoma (23 cases), adenosquamous carcinoma (3 cases), endometrial mucinous adenocarcinoma (1 case), serous papillary adenocarcinoma (1 case) and carcinosarcoma (2 cases).

### Qualitative analysis of image quality

**Fig. 3:**
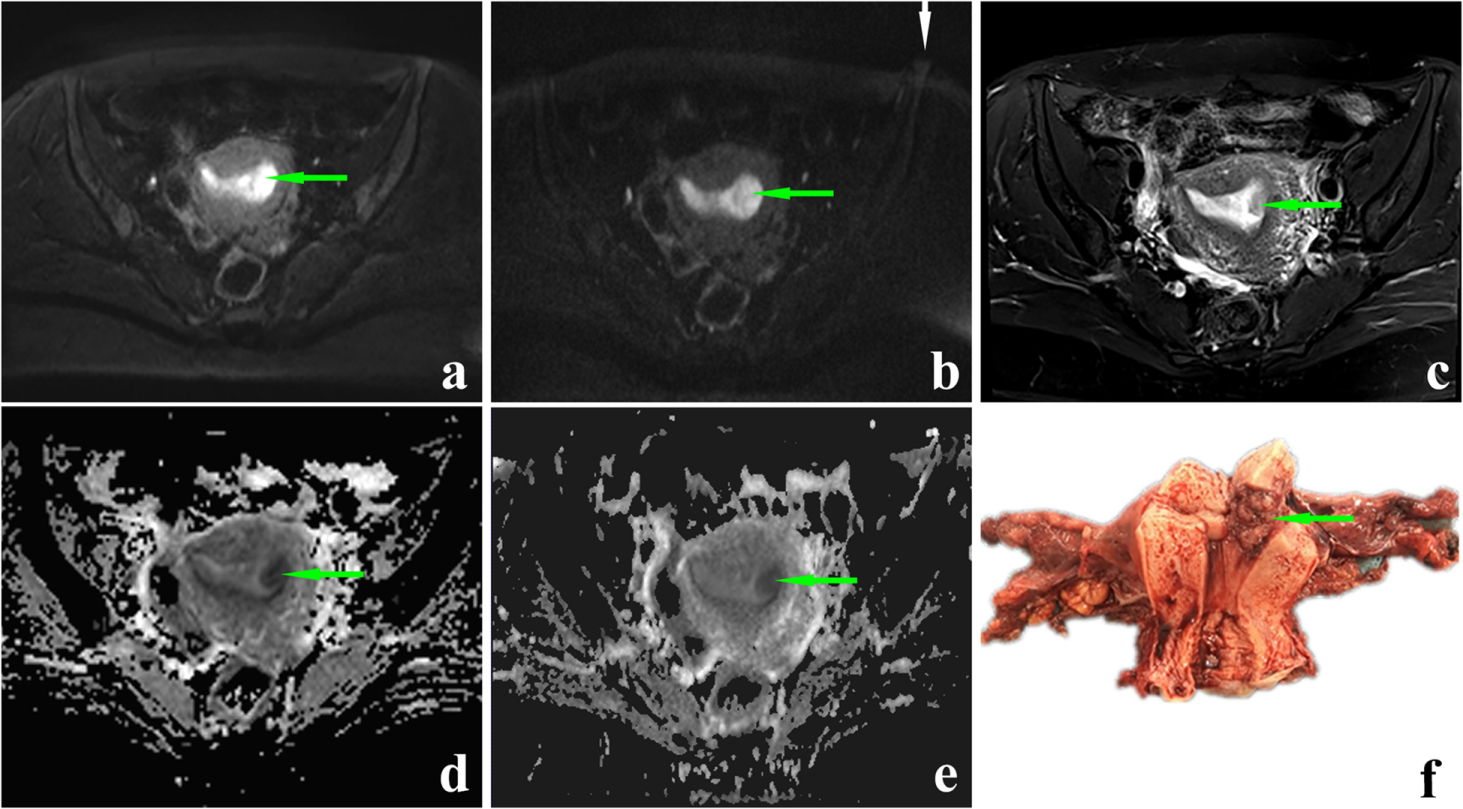
A 46-year-old patient with IA stage endometrial cancer. The endometrial cancer lesion on axial RESOLVE DWI image (**a**, green arrow), SS-EPI DWI image (**b**, green arrow), T2-weighted image (**c**, green arrow), ADC map corresponding to RESOLVE DWI sequence (**d**, green arrow) and ADC map corresponding to SS-EPI DWI sequence (**e**, green arrow). The b value of all the images is 1000s/mm^2^. The lesion can be seen more clearly on (a) than on (b). Geometric distortion can be seen on (b) (white arrow). **f** The postoperative view of the specimen. The invasion depth of endometrial carcinoma lesion was less than half of the thickness of myometrium

**Fig. 4:**
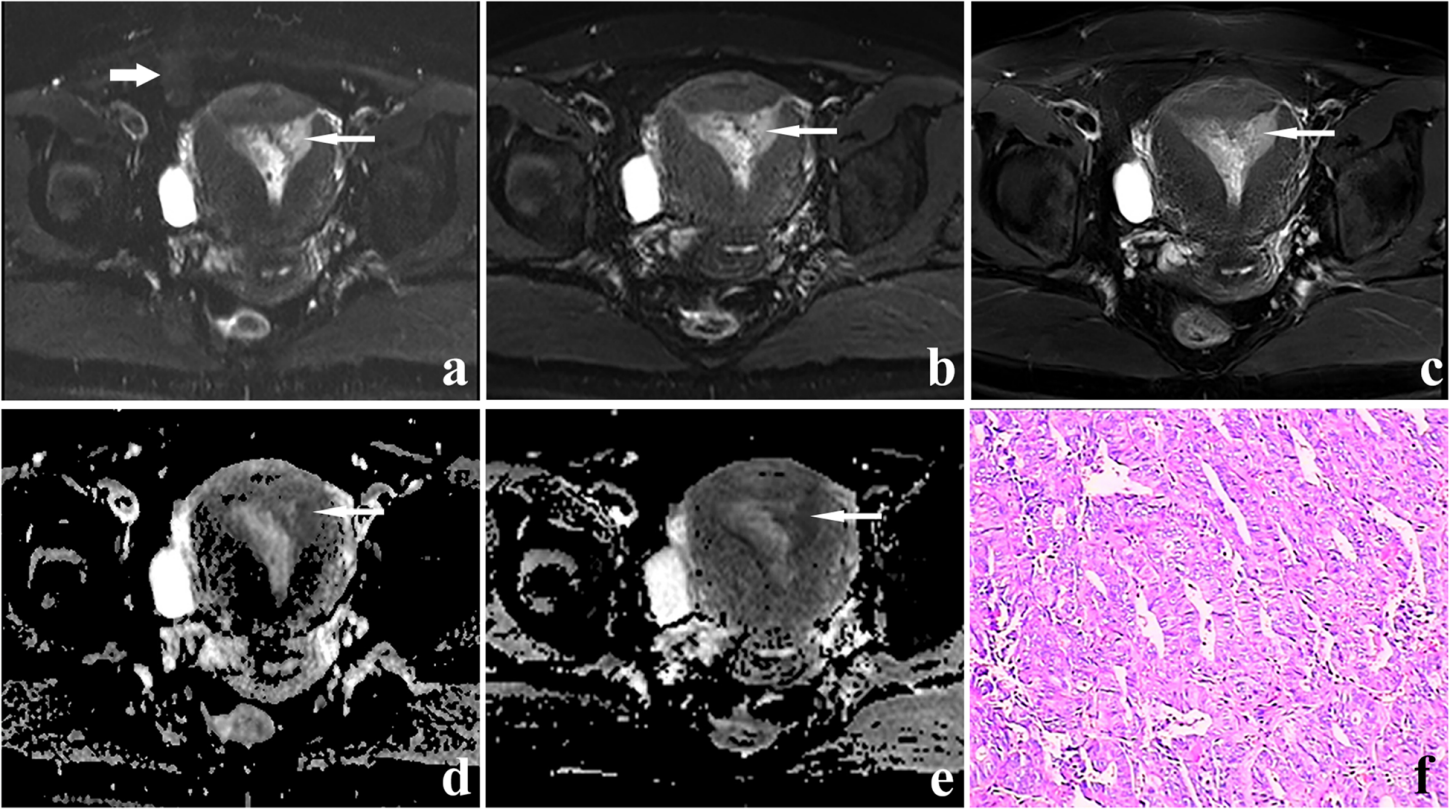
A 47-year-old patient with stage IA endometrial carcinoma. The lesion (thin white arrow) on axial SS-EPI DWI image (**a**), RESOLVE DWI image (**b**), T2-weighted image (**c**), ADC map corresponding to SS-EPI DWI (**d**) and ADC map corresponding to RESOLVE DWI (**e**). The b value of all the images is 1000s/mm^2^. Artifacts were distinct on (a) (thick arrow). The hyperintense endometrial cancer lesion can be seen more clearly on (b) than on (a). **f** H&E staining (× 100 magnification). The pathological diagnosis was well-differentiated endometrioid adenocarcinoma

According to the scan sequence, the obtained data were divided into two groups: RESOLVE DWI and SS-EPI DWI. The qualitative scores of two evaluators in Table 3 showed each aspect in the RESOLVE DWI group was better than that in the SS-EPI DWI group (all *P* < 0.001). Interobserver agreement of the depiction of geometric distortion, image blurring, ghosting artifacts, lesion conspicuity and overall image quality were good or excellent in two DWI sequences (RESOLVE: 0.671, 0.674, 0.83, 0.796, 0.835; SS-EPI: 0.634, 0.644, 0.947, 0.873, 0.668, Supplementary Table 4). Examples of the advantages of RESOLVE DWI were presented in Figs. 3 and 4.

**Table 3:** Comparison of qualitative scores of DWIs using SS-EPI and RS-EPI technique

parameter	Reader 1(Mean Score ± SD)		*P* Value ^a^	Reader 2(Mean Score ± SD)		*P* Value ^a^
	SS-EPI	RS-EPI		SS-EPI	RS-EPI	
Geometric distortion	4.63 ± 0.556	5.70 ± 0.466	< 0.001	4.80 ± 0.551	5.77 ± 0.430	< 0.001
Image blurring	3.33 ± 0.711	4.70 ± 0.535	< 0.001	3.00 ± 0.743	4.53 ± 0.571	< 0.001
Ghosting artifacts	3.47 ± 0.629	4.97 ± 0.320	< 0.001	3.43 ± 0.626	4.93 ± 0.254	< 0.001
lesion conspicuity	3.53 ± 0.937	4.87 ± 0.730	< 0.001	3.43 ± 1.006	4.80 ± 0.847	< 0.001
Overall image quality	4.50 ± 1.306	7.27 ± 0.868	< 0.001	4.37 ± 1.273	7.10 ± 0.960	< 0.001

*DWI* diffusion-weighted imaging, *SS-EPI* single-shot echo-planar imaging, *RS-EPI* readout-segmented echo-planar imaging, *SD* standard deviation

### Quantitative analysis of image quality

The average values of SNR for SS-EPI DWI and RESOLVE DWI were 51.37 ± 18.37 and 279.46 ± 109.48, respectively. The SNR values of the RESOLVE DWI were higher than those of SS-EPI DWI (*P*<0.001, 95% CI: 191.28–264.89, Fig. 2). The average values of CNR were significantly higher for RESOLVE DWI (176.12 ± 72.72) than those for SS-EPI DWI (34.72 ± 15.36) (*P* < 0.001, 95% CI: 117.39–165.41, Fig. 2). The average ADC values for SS-EPI DWI and RESOLVE DWI were (0.920 ± 0.055) × 10^− 3^ mm^2^/s and (0.890 ± 0.052) × 10^− 3^ mm^2^/s, respectively. No statistical difference in ADC value was observed between SS-EPI DWI and RESOLVE DWI (*P* = 0.261).

### Myometrial invasion of endometrial carcinoma

Diagnostic test evaluation indexes for judging myometrial invasion were summarized in Table 4. Only the sensitivities of assessing intramucosal lesion by the two scanning methods were the same. The sensitivity of assessing myometrial invasion in the other two cases by RESOLVE DWI were both higher than those by SS-EPI DWI. The specificity, accuracy, PPV and NPV of estimating myometrial invasion in three cases by RESOLVE DWI were all higher than those by SS-EPI DWI in endometrial carcinoma. We further analyzed 95% confidence interval of sensitivity and PPV. We found that RESOLVE DWI was valuable in judging <50% superficial invasion. The positive likelihood ratios of RESOLVE DWI group in three cases were all higher than those of the SS-EPI DWI group. The negative likelihood ratios of the RESOLVE DWI group in three cases were all lower than those of the SS-EPI DWI group.

**Table 4:** Diagnostic test evaluation indexes for judging myometrial invasion by RESOLVE DWI and SS-EPI DWI.

Parameters	Intramucosal lesion		< 50% superficial invasion		≥50% myometrial invasion	
	Resolve DWI	SS-EPI DWI	Resolve DWI	SS-EPI DWI	Resolve DWI	SS-EPI DWI
Sensitivity (95% confidence interval)	4/6 (66.67%) (0.289, 1.044)	4/6 (66.67%) (0.289, 1.044)	14/18 (77.78%) (0.586, 0.970)	6/18 (33.33%) (0.116, 0.551)	4/6 (66.67%) (0.289, 1.044)	1/6 (16.67%) (−0.132, 0.465)
Specificity	22/24 (91.67%)	16/24 (66.67%)	8/12 (66.67%)	5/12 (41.67%)	22/24 (91.67%)	20/24 (83.33%)
Accuracy	26/30 (86.67%	20/30 (66.67%)	22/30 (73.33%)	11/30 (36.67%)	26/30 (86.67%)	21/30 (70%)
Positive predictive value (95% confidence interval)	4/6 (66.67%) (0.289, 1.044)	4/12 (33.33%) (0.067, 0.600)	14/18 (77.78%) (0.617, 0.938)	6/13 (46.15%) (0.191, 0.733)	4/6 (66.67%) (0.289, 1.044)	1/5 (20%) (−0.151, 0.551)
Negative predictive value	22/24 (91.67%)	16/18 (88.89%)	8/12 (66.67%)	5/17 (29.41%)	22/24 (91.67%)	20/25 (80%)
Positive likelihood ratio	8.004	2.000	2.333	0.571	8	1
Negative likelihood ratio	0.364	0.500	0.333	1.600	0.364	1

*RESOLVE* readout segmentation of long variable echo-trains, *DWI* diffusion-weighted imaging, *SS-EPI* single-shot echo-planar imaging

### Stage of endometrial carcinoma

**Fig. 5:**
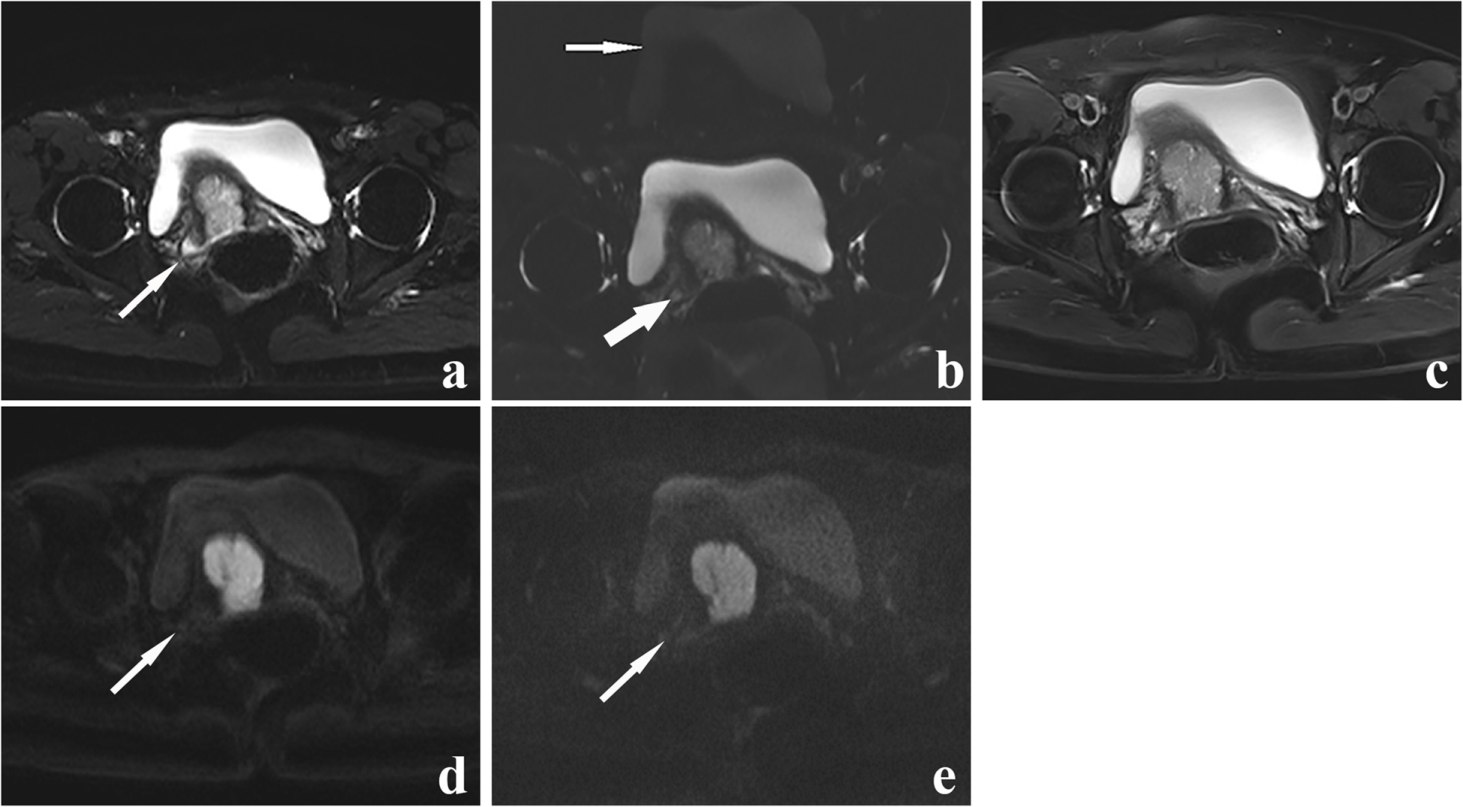
A 54-year-old woman with endometrial cancer (endometrioid carcinoma, moderately differentiated, superficial myometrial invasion). **a** An axial RESOLVE DWI image (b = 0 s/mm^2^). **b** an axial SS-EPI DWI image (b = 0 s/mm^2^). **c** an axial T2WI image. **d** An axial RESOLVE DWI image (b = 1000s/mm^2^). **e** an axial SS-EPI DWI image (b = 1000s/mm^2^). The right side of the uterus is presented as a hyperintense signal on (a) (white arrow). (b) showing only a slightly higher signal intensity in the lesion (thick arrow). This suggested endometrial cancer on the right side of the uterus. The artifacts were seen on (b) (thin white arrow). As indicated by the white arrow, the lesion can be seen more clearly in (d) than in (e). Therefore, this case of stage IIIB endometrioid adenocarcinoma was accurately diagnosed by RESOLVE DWI

The accuracy of preoperative staging by RESOLVE DWI and SS-EPI DWI were 86.67% (26/30) and 66.67% (20/30) in endometrial cancer, respectively (Table 5). The results showed no significant difference in accuracy staging was between the two methods (*P* = 0.125). 4 cases were wrongly staged by RESOLVE DWI and 10 cases were by SS-EPI DWI. The details were as follows: IA misdiagnosed as IB (RESOLVE: 1 case; SS-EPI: 3 cases), IB misdiagnosed as IA (RESOLVE: 1; SS-EPI: 3), II misdiagnosed as IA (SS-EPI:1), IIIA misdiagnosed as IB (RESOLVE: 1; SS-EPI: 1), and IIIB case misdiagnosed as II (RESOLVE: 1; SS-EPI: 2) (Supplementary Table 5). One case of stage IIIB endometrioid adenocarcinoma was accurately diagnosed by RESOLVE DWI (shown in Fig. 5).

**Table 5:** The staging of 30 endometrial cancer patients by RESOLVE and SS-EPI DWI technology

FIGO Stage	N	The number of accurate staging	
		Resolve DWI	SS-EPI DW
IA	21	20	18
IB	4	3	1
II	1	1	0
IIIA	1	0	0
IIIB	2	1	0
IIIC	0	0	0
IV	1	1	1

*RESOLVE* readout segmentation of long variable echo-trains, *SS-EPI* single-shot echo-planar imaging, *DWI* diffusion-weighted imaging, *FIGO* International Federation of Gynecology and Obstetrics

## Discussion

Accurate preoperative evaluation of endometrial cancer lesions is very important for conservative treatment [9]. This also helps choose the appropriate approach of surgery and monitoring treatment effect. Our study confirmed that RESOLVE DWI technology can provide high-quality MR images of endometrial cancer lesions. The high-quality images are helpful for precise assessment of myometrial invasion of endometrial cancer.

The increased cellularity of endometrial cancer causes restricted diffusion, which leads to increased signal on the DW image and a reduced ADC value [25]. Compared with T1WI-DCE, SS-EPI DWI shows great accuracy, sensitivity and specificity in diagnosing myometrial invasion of endometrial carcinoma [26]. The fusion of SS-EPI DWI and T2WI images are also helpful for anatomical location and improve the accuracy of myometrial invasion of endometrial carcinoma. However, the distortion and blurring that are proportional to the matrix size in SS-EPI DWI still limit the application of this technique for the acquisition of high-resolution images [27]. RESOLVE is a new RS-EPI technique and integrates this method with two-dimensional navigator correction to correct phase errors. The MR image quality is a key point of detecting and evaluating a lesion. So we evaluated the two kinds of DWI images in terms of geometric distortion, image blurring, artifacts, lesion display, and overall image quality. Our study demonstrated that RESOLVE DWI can obtain high-resolution images with less geometric distortion and artifacts. Another study of pelvic diseases had similar results. Thirty patients (containing three for cervical/endometrial cancer) underwent pelvic MRI with both SS-EPI and RS-EPI DWI. The study confirmed that RS-EPI DWI images showed better image quality compared to the SS-EPI technique at 3 T [20].

Quantitative evaluation of DWI image quality includes SNR, CNR and ADC. The SNR is an important quantity used to describe the performance of an MRI system. The most commonly used technique is to determine the signal intensity in the tissue of interest and to measure the noise intensity in the image background. Gassert FT et al. [28] and Gourtsoyianni S et al. [29] in their research applied this method, which is consistent with ours. The DWI images produced by the RESOLVE sequence showed higher SNR and CNR values compared with SS-EPI in endometrial carcinoma. This suggested that SI difference among images of different tissues was more obvious in RESOLVE DWI images. This feature may be convenient for identifying lesions. ADC value is related to the acquisition technique, including magnetic field strength, gradient performance, respiratory compensation technology, and the choice of b-values [30–33]. The ADC values of endometrial cancer lesions had no statistical difference between RESOLVE and SS-EPI DWI images in our study. This indicated that RESOLVE DWI technology had no effect on the ADC values. In fact, RESOLVE DWI had advantages in measuring ADC value in other lesions. Higher specificity and better reproducibility of ADC measurements were found for coronal RESOLVE DWI in acute optic neuritis patients [34]. RESOLVE offered more accurate ADC values of sinonasal lesions than SS-EPI [13].

The results of the quantitative and qualitative analysis indicate that the image quality of RESOLVE DWI is better than that of SS-EPI DWI. The technique of RESOLVE DWI has proven to provide better detection and image quality in rectal, prostate, kidney, neck, and breast compared with SS-EPI DWI [15, 16, 18, 35, 36]. Our study also supports this conclusion.

The RESOLVE DWI typically is used to get higher resolution images. Our study found RESOLVE DWI images were more helpful to judge myometrium invasion in endometrial cancer. Although only the data in 95% confidence interval of sensitivity show that RESOLVE DWI is valuable in judging <50% superficial invasion. The values of specificity, PPV, NPV, and positive likelihood ratio in the RESOLVE DWI group were all higher than those in the SS-EPI DWI group. This may be related to our small sample study. The depth of myometrial invasion represents a prognostic factors in endometrial cancer, correlating with lymph node metastases and overall patient survival [37, 38]. The lymph node metastasis rate of the FIGO IA stage and IB stage was 3 and 46% respectively [39]. Tumor invasion to greater than 50% of the myometrial thickness translates to a six to seven times greater risk of pelvic and para-aortic lymph node metastases [40]. Therefore, accurate preoperative assessment of myometrial invasion by RESOLVE DWI is crucial to the choice of surgical approach and prognostic evaluation in endometrial cancer.

Errors in the assessment of myometrial invasion can occur in larger polypoid tumors, leiomyomas, congenital anomalies and very small atrophic uterus [41]. Compared with SS-EPI, RESOLVE DWI provided significantly better imaging quality and comparable diagnostic performance in detection of the depth of myometrial invasion. These features may be helpful for preoperative tumor staging. A study on preoperative staging of 68 patients with rectal cancer showed that the accuracy, sensitivity and specificity of RESOLVE DWI were higher than those of SS-EPI DWI [42]. RESOLVE DWI has clinical significance value in preoperative staging of rectal cancer and appropriate treatment options. Although we did not find the statistical differences between the two DWI technologies in clinical staging, RESOLVE DWI showed the potential benefits of clinical staging. A stage IIIB endometrial adenocarcinoma patient was correctly staged with RESOLVE DWI, while was underestimated with SS-EPI DWI in our study.

There were several limitations in the study. First, the image quality of RESOLVE DWI and SS-EPI DWI was evaluated only in the axial plane. The combination of the sagittal and coronal images will make the results more accurate. Secondly, we found that the MRI imaging of endometrial carcinoma lesions could not be completely avoided some affecting factors. These factors include the size of endometrial cancer lesions, secretion of endometrial mucus, and the peristalsis of the intestinal canal. We need to expand the sample size, spend more time studying sequence conditions, and prepare patients for the intestinal tract to reduce the impact of these factors. Finally, the echo times of the two DWI sequences are significantly different (reflected in Table 1), because the 3.0 T Siemens MRI scanner limits the settings. Thus, this difference prevents us from fairly comparing the SNR values of the two sequences. In addition, estimating the noise from the background requires a spatially homogeneous distribution of noise over the whole image. Although we adopt the method of measuring 3 times and taking the average, it may be difficult to avoid the error caused by the uneven lesion. Regardless of the above-mentioned limitations, we believe that the RESOLVE DWI has further advantages over the SS-EPI DWI in the evaluation of endometrial carcinoma.

## Conclusion

RESOLVE DWI can provide high quality images of endometrial carcinoma, which is helpful for the accurate assessment of myometrial invasion in endometrial carcinoma. The precise estimate will be helpful to select the suitable endometrioid adenocarcinoma patients for conservative treatment and monitoring the effects. It is also helpful to select the appropriate operative type and reduce the occurrence of surgical complications for the endometrial cancer patients [43].

## Supplementary information

**Additional file 1: Supplementary Table 1.** FIGO Staging with Corresponding MR imaging.

**Additional file 2: Supplementary Table 2.** MRI criteria for the assessment of myometrial invasion.

**Additional file 3: Supplementary Table 3.** The operation methods in 30 patients.

**Additional file 4: Supplementary Table 4.** Correlation analysis of qualitative scores between two observers.

**Additional file 5: Supplementary Table 5.** Comparison of SS-EPI DWI and RESOLVE DWI in diagnosis of myometrial invasion and pathological results of endometrial carcinoma.

## Data Availability

The datasets used and/or analyzed during the current study are available from the corresponding author on reasonable request.

## Abbreviations

ADC: Apparent diffusion coefficient
CNR: Contrast to noise ratio
DW-MRI: Diffusion-weighted MRI
DCE-MRI: Dynamic contrast-enhanced MRI
DWI: Diffusion-weighted imaging
FIGO: International Federation of Obstetrics and Gynecology
Gd-DTPA: Gadopentetate Dimeglumine
MRI: Magnetic resonance imaging
NPV: Negative predictive value
PPV: Positive predictive value
RS-EPI: Readout-segmented echo-planar imaging
RESOLVE DWI: Readout segmentation of long variable echo-trains diffusion-weight imaging
ROI: Region of interest
SS-EPI: Single-shot echo-planar imaging
SNR: Signal to noise ratio
T1WI: T1-weighted imaging
T2WI: T2-weighted imaging
TSE: Turbo spin echo
