# Supplementary material for: High resolution diffusion-weighted imaging with readout segmentation of long variable echo-trains for determining myometrial invasion in endometrial carcinoma
Source: Cancer Imaging. 2020 Sep 21;20:66. doi: 10.1186/s40644-020-00346-7 (PMC7507745; doi:10.1186/s40644-020-00346-7)
Supplement: Supplementary file 1 — Additional file 1: Supplementary Table 1. FIGO Staging with Corresponding MR imaging. [file 40644_2020_346_MOESM1_ESM.docx]

Supplementary Table 1 FIGO Staging with Corresponding MR imaging

| FIGO Stage | Description | MR imaging | |
| --- | --- | --- | --- |
| Ⅰ | Tumor confined to corpus uteri |  |  |
| ⅠA | ≤50% of myometrial depth | Abnormal signal intensity extends into the ≤50% of myometrium | 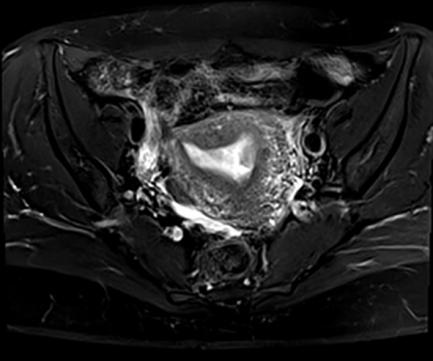 |
| ⅠB | >50% of myometrial depth | Abnormal signal intensity extends into >50% of myometrium | 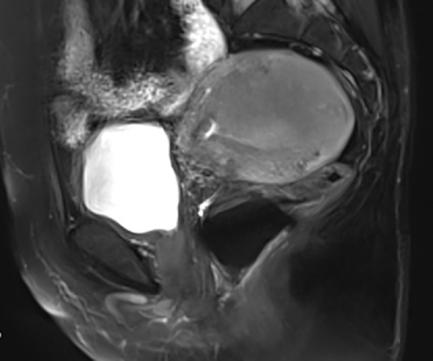 |
| Ⅱ | Tumor invades cervical stroma but does not extend beyond uterus | Disruption of hypointense stroma by tumor | 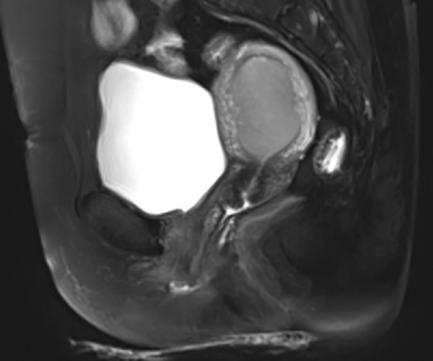 |
| Ⅲ | Local and/or regional spread of tumor |  |  |
| ⅢA | Tumor invades serosa of corpus uteri and/or adnexa | Disruption of continuity of outer myometrium | 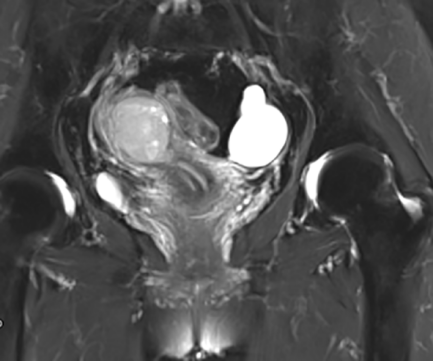 |
| ⅢB | Vaginal and/or parametrial involvement | Segmental loss of hypointense vaginal wall | 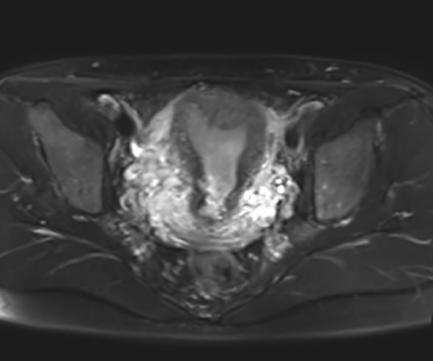 |
| ⅢC | Metastases to pelvic and/or paraaortic lymph nodes | Regional or paraaortic nodes |  |
| ⅢC1 | Positive pelvic nodes | Node size 10 mm, irregular contour, similar signal intensity to that of primary tumor | 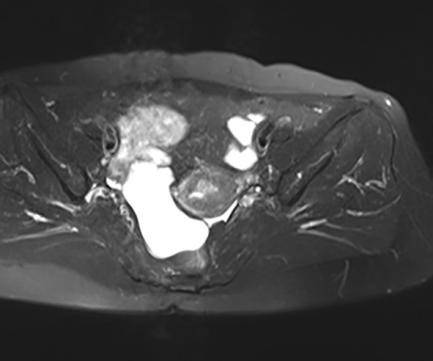 |
| ⅢC2 | Positive paraaortic lymph nodes |  | 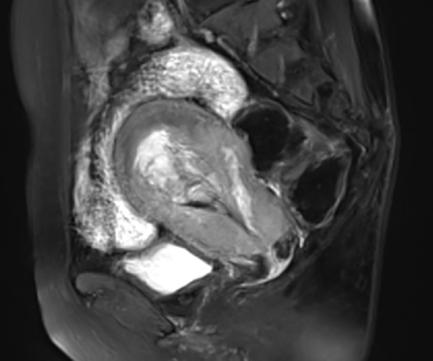 |
| Ⅳ | Tumor invades bladder and/or bowel mucosa; distant metastases may be  present |  |  |
| ⅣA | Tumor invasion of bladder and/or bowel mucosa (biopsy proven) | Abnormal signal intensity disrupts normal hypointense muscle and invades bladder and/or rectal mucosa; Bullous edema does not indicate stage ⅣA | 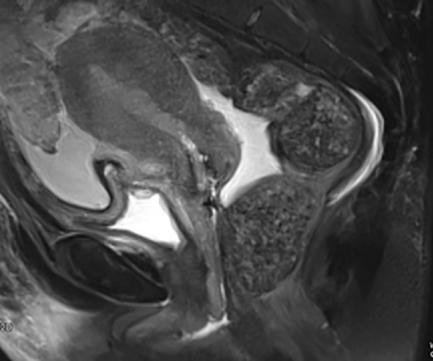 |
| ⅣB | Distant metastases, including intraabdominal metastases and/or inguinal lymph nodes | Tumor in distant sites or organs | 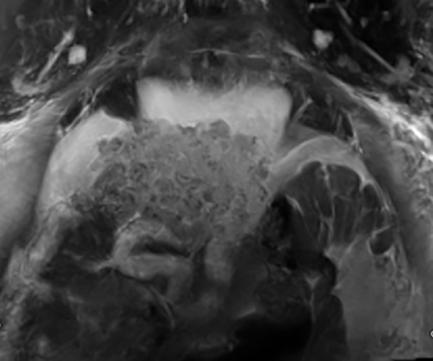 |

*FIGO* International Federation of Gynecology and Obstetrics, *MR* magnetic resonance
