# Supplementary material for: High resolution diffusion-weighted imaging with readout segmentation of long variable echo-trains for determining myometrial invasion in endometrial carcinoma
Source: Cancer Imaging. 2020 Sep 21;20:66. doi: 10.1186/s40644-020-00346-7 (PMC7507745; doi:10.1186/s40644-020-00346-7)
Supplement: Supplementary file 2 — Additional file 2: Supplementary Table 2. MRI criteria for the assessment of myometrial invasion. [file 40644_2020_346_MOESM2_ESM.docx]

Supplementary Table 2 MRI criteria for the assessment of myometrial invasion

| Myometrial invasion | MRI findings |
| --- | --- |
| Intramucosal lesion | Continuous, noninterrupted junctional zone and subendometrial enhancement |
| ＜50% superficial invasion | Disruption or irregularity of junctional zone and subendometrial  enhancement |
| ＞50% deep invasion | Complete disruption of junctional zone, subendometrial  enhancement and signal intensity of tumor extends into outer half of myometrium |

*MRI* magnetic resonance imaging
