# Supplementary material for: High resolution diffusion-weighted imaging with readout segmentation of long variable echo-trains for determining myometrial invasion in endometrial carcinoma
Source: Cancer Imaging. 2020 Sep 21;20:66. doi: 10.1186/s40644-020-00346-7 (PMC7507745; doi:10.1186/s40644-020-00346-7)
Supplement: Supplementary file 3 — Additional file 3: Supplementary Table 3. The operation methods in 30 patients. [file 40644_2020_346_MOESM3_ESM.docx]

Supplementary Table 3 The operation methods in 30 patients

| methods of operation | Surgical approach | Number of cases |
| --- | --- | --- |
| modified radical hysterectomy +bilateral salpingooophorectomy+ pelvic nodal dissection with or without para-aortic nodal dissection | laparoscopy | 15 |
|  | laparotomy | 1 |
| hysterectomy + bilateral salpingooophorectomy + pelvic nodal dissection with or without para-aortic nodal dissection | laparoscopy | 6 |
| radical hysterectomy+ bilateral salpingooophorectomy + pelvic nodal dissection with para-aortic nodal dissection | laparoscopy | 1 |
| hysterectomy + bilateral salpingooophorectomy | laparoscopy | 2 |
|  | laparotomy | 1 |
| modified radical hysterectomy + bilateral salpingooophorectomy | laparoscopy | 1 |
|  | laparotomy | 1 |
| radical hysterectomy + bilateral salpingooophorectomy + pelvic nodal dissection + omentum resection | laparotomy | 1 |
| hysterectomy + bilateral salpingooophorectomy + omentum resection +resection of metastatic focus | laparotomy | 1 |
