# Supplementary material for: High resolution diffusion-weighted imaging with readout segmentation of long variable echo-trains for determining myometrial invasion in endometrial carcinoma
Source: Cancer Imaging. 2020 Sep 21;20:66. doi: 10.1186/s40644-020-00346-7 (PMC7507745; doi:10.1186/s40644-020-00346-7)
Supplement: Supplementary file 4 — Additional file 4: Supplementary Table 4. Correlation analysis of qualitative scores between two observers. [file 40644_2020_346_MOESM4_ESM.docx]

Supplementary Table 4 Correlation analysis of qualitative scores between two observers

| parameters | SS-EPI DWI | | RESOLVE DWI | |
| --- | --- | --- | --- | --- |
|  | r_s_^a^ | P Value | r_s_^a^ | P Value |
| geometric distortion | 0.634 | ＜0.001 | 0.671 | ＜0.001 |
| image blurring | 0.644 | ＜0.001 | 0.674 | ＜0.001 |
| ghosting artifacts | 0.947 | ＜0.001 | 0.830 | ＜0.001 |
| lesion conspicuity | 0.873 | ＜0.001 | 0.796 | ＜0.001 |
| overall image quality | 0.668 | ＜0.001 | 0.835 | ＜0.001 |

*SS-EPI* single shot echo-planar imaging, *DWI* diffusion-weighted imaging, *RESOLVE* readout segmentation of long variable echo trains

Note: little or no relationship if 0≤ r_a_ <0.2, fair if 0.2≤ r_a_ <0.4, moderate if 0.4≤ r_a_ <0.6, good if 0.6≤ r_a_ <0.8, and excellent if 0.8≤ r_a_
