# Supplementary material for: High resolution diffusion-weighted imaging with readout segmentation of long variable echo-trains for determining myometrial invasion in endometrial carcinoma
Source: Cancer Imaging. 2020 Sep 21;20:66. doi: 10.1186/s40644-020-00346-7 (PMC7507745; doi:10.1186/s40644-020-00346-7)
Supplement: Supplementary file 5 — Additional file 5: Supplementary Table 5. Comparison of SS-EPI DWI and RESOLVE DWI in diagnosis of myometrial invasion and pathological results of endometrial carcinoma. [file 40644_2020_346_MOESM5_ESM.docx]

Supplementary Table 5 Comparison of SS-EPI DWI and RESOLVE DWI in diagnosis of myometrial invasion and pathological results of endometrial carcinoma

| Inspection method | Muscular infiltration | Pathological results | | | Total |
| --- | --- | --- | --- | --- | --- |
|  |  | Confined to the intima | Depth of myometrial invasion < 1 / 2 myometrial | Depth of myometrial invasion＞1 / 2 myometrial |  |
| SS-EPI DWI | Confined to the intima | 4 | 8 | 0 | 12 |
|  | Depth of myometrial invasion < 1 / 2 myometrial | 2 | 6 | 5 | 13 |
|  | Depth of myometrial invasion＞1 / 2 myometrial | 0 | 4 | 1 | 5 |
|  | Total | 6 | 18 | 6 | 30 |
| RESOLVE DWI | Confined to the intima | 4 | 2 | 0 | 6 |
|  | Depth of myometrial invasion < 1 / 2 myometrial | 2 | 14 | 2 | 18 |
|  | Depth of myometrial invasion＞1 / 2 myometrial | 0 | 2 | 4 | 6 |
|  | Total | 6 | 18 | 6 | 30 |

*SS-EPI* single shot echo-planar imaging, *DWI* diffusion-weighted imaging, *RESOLVE* readout segmentation of long variable echo trains
